# Vascular disruptive agent OXi4503 and anti-angiogenic agent Sunitinib combination treatment prolong survival of mice with CRC liver metastasis

**DOI:** 10.1186/s12885-016-2568-7

**Published:** 2016-07-26

**Authors:** Linh Nguyen, Theodora Fifis, Christopher Christophi

**Affiliations:** Department of Surgery, University of Melbourne, Austin Health, Lance Townsend Building Level 8, Studley Rd, Heidelberg, VIC 3084 Australia

**Keywords:** Combination therapy, Vascular disruptive agent, OXi4503, Hypoxia, Sunitinib, Tumor resistance

## Abstract

**Background:**

Preclinical research indicate that vascular disrupting agent (VDA) treatment induces extensive tumor death but also a systemic mobilization of bone marrow derived cells including endothelial progenitor cells (EPC) leading to revascularization and renewed growth within the residual tumor. This study investigates if combination of VDA with the anti-angiogenic agent Sunitinib increases the treatment efficacy in a colorectal liver metastases mouse model.

**Methods:**

CBA mice with established liver metastases were given a single dose of OXi4503 at day 16 post tumor induction, a daily dose of Sunitinib starting at day 14 or day 16 post tumor induction or a combination of Sunitinib given daily from day 14 or day 16 post tumor induction in combination with a single dose of OXi4503 at day 16. Treatment was terminated at day 21 post tumor induction and its effects were assessed using stereological and immunohistochemical techniques. Long term effects were assessed in a survival study.

**Results:**

Combination with long (7 day) Sunitinib treatment lead to liver toxicity but this was ameliorated in the shorter (5 day) treatment without significantly altering the effects on tumor reduction. Combination treatment resulted in significant reduction of viable tumor, reduction in tumor vasculature, reduction in tumor proliferation, increase in tumor apoptosis and prolonged mouse survival compared to control and single arm treatments. Complete tumor eradication was not achieved. Redistribution of E-cadherin and strong up regulation of ZEB1 and Vimentin were observed in the surviving tumor; indicative of epithelial to mesenchymal transition (EMT), a mechanism that could contribute to tumor resistance.

**Conclusions:**

Combination treatment significantly reduces viable tumor and prolongs animal survival. EMT in the surviving tumor may prevent total tumor eradication and could provide novel targets for a more lasting treatment.

**Electronic supplementary material:**

The online version of this article (doi:10.1186/s12885-016-2568-7) contains supplementary material, which is available to authorized users.

## Background

Tumor vasculature is essential for the growth and development of tumors beyond 1–2 mm^3^ [1, 2]. Unlike host vessels, tumor vessels are immature, unstable, leaky and deficient in pericyte and smooth muscle coverage. They undergo constant remodelling in order to supply the rapidly growing tumor with oxygen and nutrients [3]. These fundamental differences in tumor vessels compared to host vessels make them more sensitive to the effects of VDAs [4]. OXi4503 is a potent VDA which has demonstrated immediate vascular shutdown leading to ischemia and tumor necrosis [5–7]. Despite extensive tumor necrosis however OXi4503 does not completely eradicate the tumor. Incomplete destruction of the tumor is evident by the presence of a distinctive rim of viable tumor cells at the tumor periphery [5, 6]. These surviving tumor cells undergo vigorous revascularization through angiogenesis resulting in disease recurrence. Previous studies including ours have implicated tumor revascularization as a major contributor to residual disease. Combination treatments of VDAs with anti-angiogenic agents (AAs) have the potential to inhibit re-vascularization of the residual tumor and thus enhance the efficacy of the treatment [8]. Anti-angiogenic agents unlike VDAs prevent the formation of new blood vessels. Monoclonal antibodies to VEGF (Avastin®) and a number of small tyrosine kinase inhibitors targeting multiple receptors involved in angiogenesis including the VEGF receptors have been approved for clinical use. Our previous findings, together with other preclinical data support the rationale of combining VDAs with AAs as a complementary treatment strategy [9–12]. In the current study we evaluate the antitumor efficacy of combining OXi4503 with the anti-angiogenic agent, Sunitinib, in a colorectal liver metastasis (CRLM) mouse model. Sunitinib a multi-targeting inhibitor of the receptor tyrosine kinases (RTK) of VEGFR-1/-2/-3, PDGFR-α/-β, FLT3, stem cell growth factor receptor KIT and RET [13] has been approved for the treatment of advanced renal cell carcinoma, imatinib resistant or imatinib-intolerant gastrointestinal stromal tumors, and unresectable or metastatic well differentiated pancreatic neuroendocrine tumors [14–18].

## Methods

### Animals and experimental model of colorectal liver metastasis

The CRLM model used in this study was developed and fully characterized in our laboratory. The tumor grows orthotopically in a fully immunocompetent host with growth characteristics similar to human metastasis [19]. Male CBA mice (Laboratory Animal services, University of Adelaide, South Australia) were maintained in standard cages with irradiated food and water supplied ad libitum. The primary cancer cell tumor MoCR was derived from a dimethyl hydrazine (DMH)-induced primary colon carcinoma in the CBA mouse and maintained in vivo by serial passage in the flanks of CBA mice. For passage and experimentation, subcutaneous tumors were teased apart, passed through a filter, treated with EDTA and washed in PBS to make a single cell suspension. Liver metastases were induced by intrasplenic injection of 5 × 10^4^ tumor cells followed by splenectomy. Metastases are fully established by 21 days following tumor induction [19]. All animal studies were approved and conducted under the supervision and in accordance with the guidelines set by the Austin Hospital animal ethics committee.

### Drugs and treatment protocol

Treatment was administered 16 days after induction of liver metastases when tumors are well established. OXi4503 (Combretastatin A-1 trans-stilbene), kindly donated by OXiGENE (OXiGENE Inc. South San Francisco, CA), was freshly prepared by dissolving in 0.9 % sterile saline (NaCl) and protected from light. A single maximum tolerated dose of OXi4503, determined previously to be 100 mg/kg was administered via intraperitoneal injection [6]. Sunitinib malate (LC Laboratories, Boston, USA) was freshly dissolved in sterile saline and administered in daily intraperitoneal injections of 40 mg/kg as reported in other studies [20].

Three different studies were performed. In the first study animals with colorectal liver metastases were divided into four groups; The OXi4503 treated group received a single dose of 100 mg/kg of OXi4503 intraperitonealy at day 16 post tumor induction. The Sunitinib treated group received intraperitoneal daily doses of 40 mg/kg of Sunitinib malate from day 14 to 21 post tumor induction. The combination group received 40 mg/kg of Sunitinib daily from day 14 to 21 post tumor induction and additionally received a single dose of 100 mg/kg of OXi4503 at day 16 post tumor induction. The control group received daily intraperitoneal saline injections from day 14 to 21. In the second and third studies Sunitinib treatment for the single and combination arms of the study started at day 16. All other conditions were as for the first study. Tissues were collected 21 days post tumor induction in the first and second studies. The third was a survival study and once treatment was terminated at day 21 mice were monitored for health indicators and were killed when these became significant.

### Stereology

At the endpoint laparotomy was performed and the abdominal cavity examined for indications of macroscopic extra- hepatic metastases; paying close attention to the splenic bed. The liver was carefully excised and harvested. Immediately following excision liver weights were recorded. Organs were then fixed in formalin (10 %) (Sigma Aldrich, Castle Hill, NSW, Australia) for 24 h and then transferred to 70 % ethanol. Images of the whole liver were taken to examine the tumor distribution and burden load. The liver was then transversely sliced into sections of 1.5 mm thickness using a tissue fractionator. For large livers (Saline control and OXi4503 treated) every second slice was sampled for analysis. For smaller livers (Sunitinib and Sunitinib/OXi4503 treated tumors) every slice was taken for analysis due to small number of sections. Liver slices were placed on a clear plastic plate; a digital camera (Nikon Coolpix5000, E500) was used to capture the images and these were analysed using image analysis software (Image Pro Plus, Perth, Australia).

Tumors were visualised as distinctive white/cream coloured areas against the red/brown liver tissue. Each tumor outline was traced using image analysis software to determine the area occupied by the tumor. This stereology technique was used to determine the number of tumor nodules, tumor volume and the percentage of liver metastases.

### Immunohistochemistry

Formalin fixed paraffin tissue sections (4 μm) were used with an indirect peroxidase labeling technique (Envision Plus, DAKO, Australia). Following deparaffinization and rehydration, endogenous peroxidase activity was blocked with 3 % H_2_O_2_ and non-specific binding inhibited with 10 % normal goat serum (01–6201 Zymed Laboratories, USA). Heat induced epitope retrieval was used. Antigens were visualised by immunohistochemical staining using their respective antibodies diluted as follows: (CD34 1:500, AbD serotec MCA18256; Ki-67 1:100, Thermoscientific, RM-9106-S1; caspase 3 1:800, R&D AF835) and EMT markers (ZEB1 1:200, Santa Cruz sc-25388; Vimentin 1:300, Santa Cruz sc-5565; E-cadherin 1:500 Sana Cruz sc-7870; b-catenin 1:300, Santa Cruz sc-7199) Negative controls were stained by the corresponding isotype antibodies. Following primary antibody treatment, sections were incubated with a horseradish peroxidase labelled polymer secondary antibody. The antigen-antibody complex was visualized by diaminobenzene (DAB) Peroxidase Substrate Solution (DAKO, Australia). Each treatment group consisted of 5–8 animals. A minimum of ten tumors were assessed for each treatment group and from 3 to 15 images per tumor, depending on tumor size, were captured for analysis.

### Statistical analysis

All data was represented as the mean ± standard error of the mean. Statistical analysis was conducted using SPSS (Statistical Package for the Social Sciences,™ version 10, Chicago, Illinois, USA) using both parametric and non-parametric analytical tests as appropriate. All statistical tests were two-sided and a *P* value of 0.05 was considered statistically significant.

## Results

### Combination therapy has an additive effect on tumor death

A single OXi4503 treatment has profound effects immediately following treatment and it peaks by 24 h, then there is a robust re-vascularization and regrowth by day 5 post-treatment [10] and Additional file 1. In contrast continuous treatment with Sunitinib reduces the tumor mass gradually (Additional file 1).

**Fig. 1:**
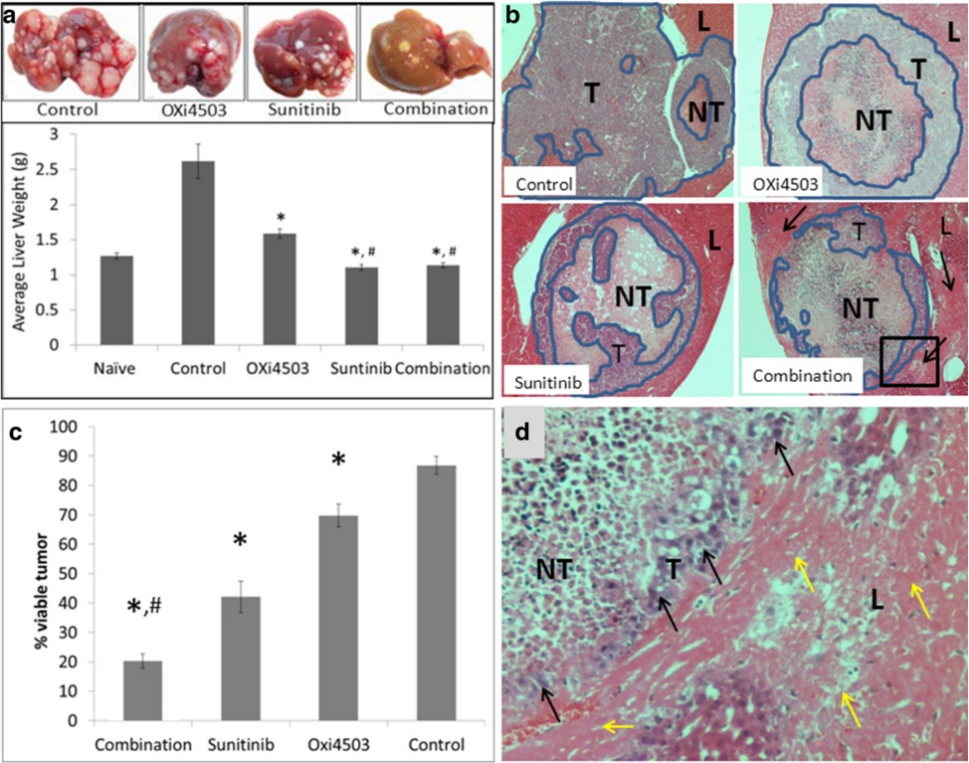
Treatment effects on CRC liver metastases. Sunitinib (40 mg/kg) was given daily from day 14 post tumor induction while OXi4503 (100 mg/kg) was given as a single dose at day 16. **a** Livers with tumor metastases (*upper panel*). Liver weights (*lower panel*) expressed as mean value ± SEM, with n ≥ 5 for each group. Significant decrease in liver weight in treatment groups compared to control (*P < 0.05). Significant decrease in liver weight in Sunitinib and combination treatments compared to naïve liver group (^#^ *P* < 0.011 and 0.007, respectively). Statistical significance determined using ANOVA with post-hoc Tukey-HSD tests. **b** H&E staining tumor/liver tissues following treatment. Scale bar = 200 μm T = Tumor, L = Liver. Live tumor areas enclosed within *blue lines. Arrows* in combination panel indicate areas of liver damage. **c** quantitation of live tumor area expressed as mean value ± SEM, with n ≥ 5 for each group. (*P < 0.01 for combination vs control or other treatments). **d** magnification of *inset* in combination panel (**b**) depicting low frequency of viable tumor cells (*black arrows*) and liver damage (*yellow arrows*). Scale bar = 50 μm

Visual examination of the livers collected at the endpoint (day 21) indicate all treatment modalities have significantly less tumor burden than the control, especially the Sunitinib and combination treatments (Fig. 1a, top panel). Livers in the combination treated group have a yellow tinge suggesting liver damage (Fig. 1a, top panel). Liver weight is commonly used as an indication of tumor burden. For all treatment regimens liver weight was significantly lower than the untreated controls (*P* < 0.05) indicating tumor death and/or inhibition of tumor proliferation (Fig. 1a, lower panel). However there was no significant difference between the liver weights of the Sunitinib treatment and the OXi4503/Sunitinib combination (Fig. 1a, lower panel). Furthermore, Sunitinib/OXi4503 combination treated livers and Sunitinib treated livers demonstrated a significantly decreased mean liver weight compared to livers from non tumor bearing animals (Fig. 1a, lower panel) (*P* = 0.007 and *P* = 0.011, respectively), again suggesting some degree of liver toxicity. Histological examination of the tumors demonstrate less live tumor remaining after the combination treatment than in either of the single arm treatments (Fig. 1b-d). In the combination treatment small patches of viable tumor are seen only in the periphery most often not forming a continuous live rim (Fig. 1b). Quantitation of live tumor area demonstrate significantly less live tumor in the combination treatment compared to single arm treatments and about half the live tumor area compared to Sunitinib (*P* < 0.01, Fig. 1c). Additionally the density of viable tumor cells in the combination treatment is less than in the single arm treatments and often only detached single viable cells can be seen, (arrows, Fig. 1d) indicating that the combination effect on tumour is more extensive than the area enumeration shows. These results indicate that measurement of viable tumor is a better indication of treatment efficacy. Further histological examination of the tissues confirms liver damage in the combination treatment as suggested from the visual liver appearance and the reduced liver weight. Extensive areas of liver tissue adjacent to tumor nodules (arrows in Fig. 1b and d) show loss of the classical sinusoidal architecture and cells display loss of nuclei and visible cell boundaries. Some areas of liver tissue distant to the tumor also display damage (not shown). Histological examination of the Sunitinib only treatment displayed limited liver damage in only two of the animals (not shown).

### OXi4503 combination with shorter Sunitinib treatment confers enhanced efficacy with no apparent liver toxicity

**Fig. 2:**
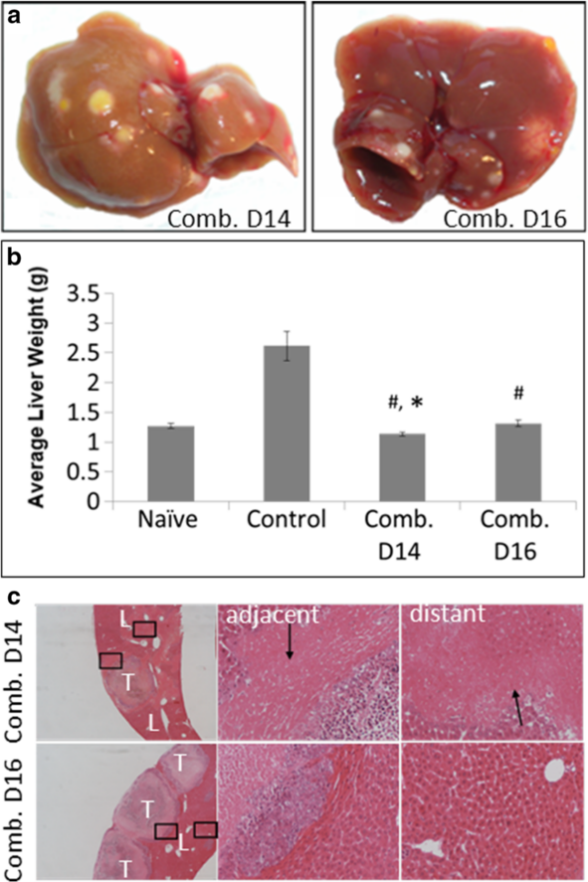
Shorter Sunitinib treatment ameliorates liver toxicity. Sunitinib (40 mg/kg) was given daily from either day 14 or day 16 post tumor induction. OXi4503 (100 mg/kg) was given as a single dose at day 16. **a** Livers with tumor metastases (No yellow tinge in Day 16 combination treatment). **b** Liver weight of treated vs control (^#^ *P* < 0.05). Significant decrease in liver weight of Day 14 combination treatment vs naïve (non tumor bearing) liver (^*^ *P* < 0.011) but not in Day16. Data is expressed as mean value ± SEM, with n ≥ 5 for each group. Statistical significance determined using ANOVA with post-hoc Tukey-HSD tests. **c** H&E staining tumor/liver tissues following treatment. L = liver, T = tumor. First column Scale bar = 200 μm Second and third column images represent *insets* from first column images depicting liver damage (*arrows*) in liver tissue adjacent and distant to tumors in Day 14 but not in Day 16 combination treatment. Scale bar = 50 μm

In view of the observed liver toxicity, in the second study Sunitinib was administered on day 16 post tumor induction, the same time as OXi4503. Visual examination of the liver did not demonstrate the yellow colouring seen in the combination treatment when Sunitinib treatment commenced on day 14 (Fig. 2a) Livers weights from the combination treatment commencing on day 16 were not significantly different to non-tumor bearing livers (Fig. 2b) unlike the results in the first study. Furthermore upon histological examination no liver damage was observed in this group with liver tissue immediate and distal to the tumor remaining morphologically unchanged with large nuclei and the classical sinusoid architecture intact (Fig. 2c). The effectiveness of starting Sunitinib treatment on day 16 post tumor induction compared to day 14 post tumor induction was measured by calculating the percentage of viable rim following treatment. Quantitation of the percentage of viable rim following the two different treatment regimens revealed an increasing trend for reduced efficacy in the shorter treatment but did not reach significance (*P* = 0.105, result not shown). The results suggest liver cytotoxicity in the combination treatment could be limited by changing the treatment schedule while still maintaining increased efficacy compared to single arm treatments.

### OXi4503/Sunitinib combination treatment exerts anti-angiogenic effects on tumors

**Fig. 3:**
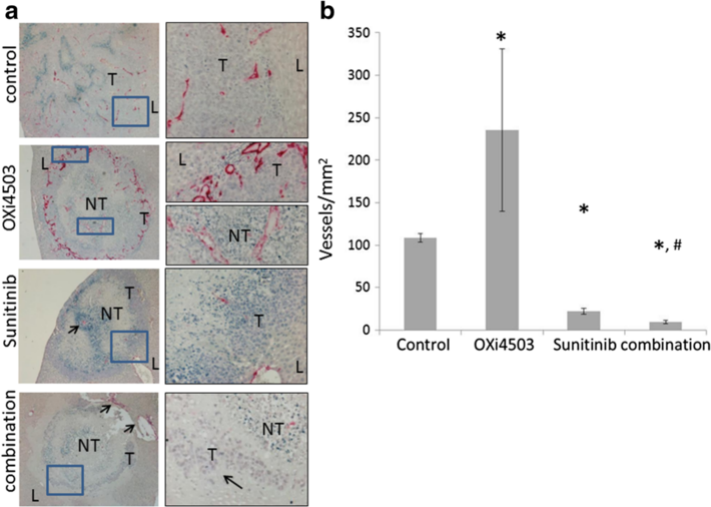
Differences in blood vessel density following different treatment regimens. **a** Formalin fixed liver sections with CRC liver metastases were stained with antibodies to CD34 (staining of endothelial cells). First column Scale bar = 200 μm. Second column depicts magnified inset regions of first column images. Scale bar = 50 μm. T = live tumor, NT = necrotic tumor. Second *inset* in the OXi4503 treatment indicates central tumor vessels recovering ahead of live tumor repopulation. *Arrow* in the Sunitinib treatment indicates intact central vessel surrounded with live tumor cells. *Arrows* in the combination treatment show liver damage. **b** Quantification of CD34 positive vessels. Data is expressed as mean value ± SEM, with n ≥ 5 for each group. Data was not normally distributed and non-parametric analysis was performed and statistical significance determined using Mann-Whitney *U* test. (OXi4503 vs control *P < 0.001, Sunitinib and combination vs control *P < 0.001 and Sunitinib vs combination ^#^ *P* = 0.001)

By day five after treatment (endpoint in this study) vigorous revascularization occurs in the residual tumor in the OXi4503 treated animals (*P* < 0.001) compared to control, (Fig. 3). Interestingly the original vessels in the necrotic centre appear to be repopulating with CD34 staining endothelial cells, despite absence of viable tumor in that area (Fig. 3 OXi4503 second inset). In contrast in the Sunitinib treatment and especially in the combination treatment, tumor vessels are significantly reduced at this endpoint compared to untreated control and OXi4503 treated groups (*P* < 0.001) (Fig. 3). In the Sunitinib treated group, major vessels are seen mainly in the tumor host interface but a few major vessels persist in central tumor regions (Fig. 3, Sunitinib, arrow). Additionally there is a marked reduction in smaller vessels within the viable tumor areas (Fig. 3). In the combination treatment vessels are only visible at the tumor edge, being most likely host vessels (Fig. 3). These results demonstrate that in the combination group Sunitinib prevents the accelerated revascularization of the residual tumor seen following a single dose of OXi4503 treatment. In addition significantly fewer tumor vessels are seen in the combination treatment compared to single arm Sunitinib treatment (*P* = 0.001).

### Combination treatment reduces live tumor load by inhibiting proliferation and increasing apoptosis

**Fig. 4:**
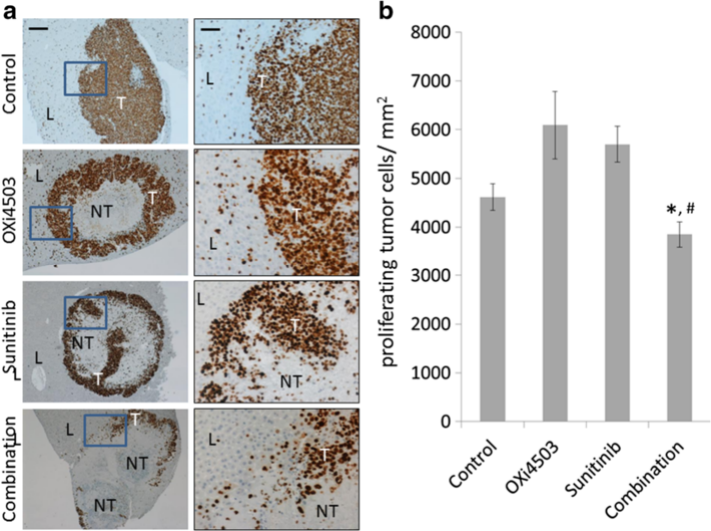
Changes in tumor proliferation following different treatment regimens. **a** Formalin fixed liver sections with CRC liver metastases were stained for the proliferation marker KI67. First column Scale bar = 200 μm. Second column represents area within the *inset* of the first column at a higher magnification. Scale bar = 50 μm. **b** enumeration of proliferating cells *P < 0.003, combination vs OXi4503 and ^#^ *P* < 0.016, combination vs Sunitinib. Data was not normally distributed and non-parametric analysis was performed and statistical significance determined using Mann-Whitney *U* test

**Fig. 5:**
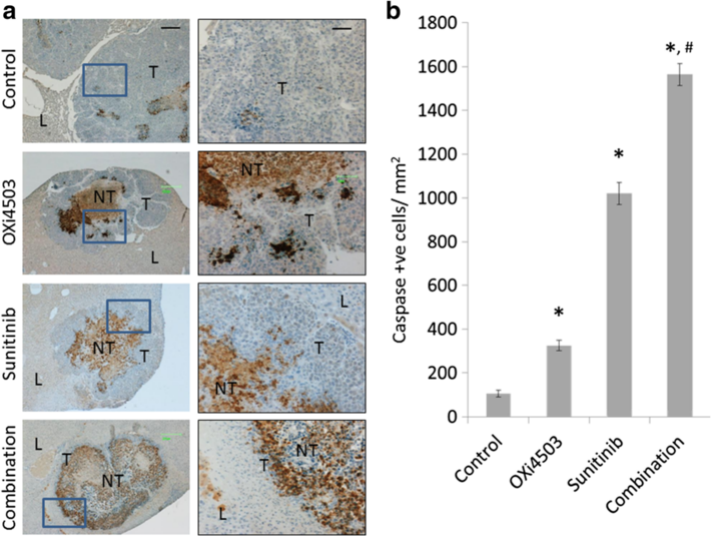
Changes in tumor apoptosis following different treatment regimens. **a** Formalin fixed liver sections with CRC liver metastases were stained for caspase 3 (apoptosis marker) First column Scale bar = 200 μm. Second column represents area within the *inset* of the first column at a higher magnification. Scale bar = 50 μm. **b** enumeration of apoptotic cells. Treatment groups vs control group (*P <0.003), Combination treated group vs OXi4503 treated (^#^ *P* <0.006) and vs Sunitinib (^#^ *P* <0.02. Data is expressed as mean value ± SEM, with n ≥ 5 for each group. Data was not normally distributed and non-parametric analysis was performed and statistical significance determined using Mann-Whitney *U* test

To determine the mechanisms of anti-tumorigenic effects of the different treatments we examined tumor proliferation and apoptosis. Staining and quantitation of Ki67 (Fig. 4a) revealed proliferation within the viable tumor areas in all groups. While live tumor areas were significantly larger in the controls and the regrowing OXi4503 group, proliferation in the OXi4503 and the Sunitinib treated groups is trending higher compared to control but not reaching significance for either treatment (Fig. 4b). In contrast the combination treatment is trending lower compared to untreated control and was significantly lower than either of single arm treatments (*P* < 0.003, combination vs OXi4503 and *P* < 0.016, combination vs Sunitinib) suggesting that reduced proliferation or at least prevention of the proliferation stimulation seen in each of the single arm treatments is one of the mechanisms for the improved efficacy in the combination treatment. Staining with Caspase 3, a marker for active apoptosis, showed increased cell apoptosis within the viable tumor regions in all treatment groups and especially in the Sunitinib and combination groups (Fig. 5a).

Apoptosis was not uniform but appeared in patches within the viable tumor regions. Enumeration of caspase positive cells (Fig. 5b) confirmed significant increase in apoptosis (treatments vs control *P* < 0.003) In addition significantly higher apoptosis occurred in the combination group compared to the OXi4503 treatment, *P* < 0.006 and the Sunitinib treatment, *P* < 0.02.

### OXi4503/Sunitinib combination treatment of liver metastases increases survival

**Fig. 6:**
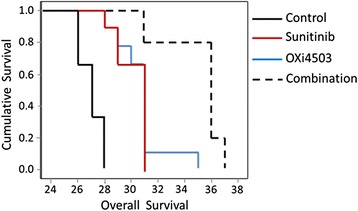
OXi4503, Sunitinib and combination treatments induce EMT in the surviving tumor cells. Formalin-fixed control and treated tumor sections were stained with antibodies to E-cadherin, ZEB1, or Vimentin. Positive expression is detected by the brown staining. Scale bar = 50 μm. L = liver, T = live tumor. NT = necrotic tumor. Images are representative for each treatment group (n ≥ 5 animals)

A survival study was undertaken to test if the enhanced tumor reduction seen in the combination treatment translates into survival advantage. Sunitinib/OXi4503 combination treatment significantly prolonged survival in tumor bearing mice compared to saline treated control, OXi4503 and Sunitinib monotherapies. Sunitinib/OXi4503 treatment Cumulative survival using the Kaplan–Meier analysis and a log rank test, demonstrate a significant difference in survival between the Sunitinib/OXi4503 combination treatment and all other treatments (Fig. 6, OXi4503/Sunitinib vs Control *P* < 0.004, vs OXi4503 *P* < 0.006, vs Sunitinib *P* < 0.005).

### Morphological changes in residual tumor cells following single and combination treatments show EMT

**Fig. 7:**
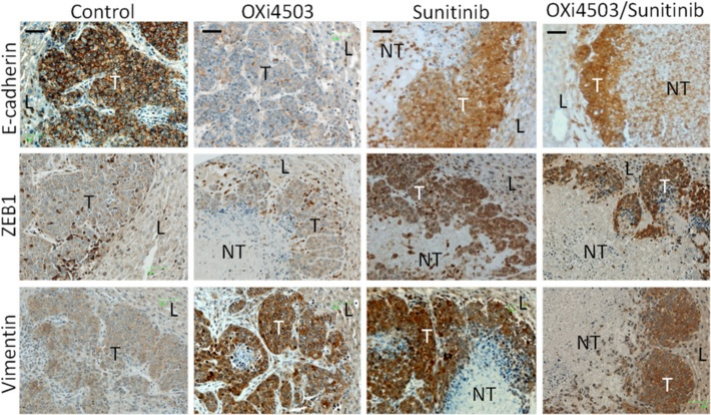
OXi4503/Sunitinib combination treatment prolongs survival of mice with CRC liver metastasis. Kaplan–Meier analysis for cancer related survival between control, OXi4503, Sunitinib and Sunitinib/OXi4503 treated groups. Data is expressed as mean value ± SEM, with *n* = 10 for each group. Sunitinib (40 mg/kg)/OXi4503 (100 mg/kg) combination treatment resulted in significant improvement in median or overall survival compared to control as found by log rank test (Sunitinib/OXi4503 vs Control *P* < 0.004, vs OXi4503 *P* < 0.006, vs Sunitinib *P* < 0.005)

While the OXi4503/Sunitinib combination was more efficacious that either single arm treatment, complete tumor eradication was not achieved. We investigated whether cells surviving the treatment become resistant by changing their morphology from epithelial to mesenchymal (Fig. 7 and Additional files 2 and 3). Untreated tumors express high levels of E-cadherin localised mainly in the cell junctions and exhibit the characteristic cobblestone staining of epithelial cells (Fig. 7 control).

ZEB1 is expressed strongly by infiltrating stroma cells that are found scattered throughout the tumor and most frequently closer to the tumor periphery and the adjacent liver tissue. Vimentin is expressed at very low levels in the control tumors but some infiltrating stroma cells stain positive (Fig. 7 and Additional file 2, control). In our previous studies we found almost complete loss of E-cadherin within an hour of OXi4503 treatment and significant up regulation of ZEB1 and Vimentin [9]. At day five post treatment (endpoint in this study) E-cadherin is expressed at significantly lower levels compared to control, ZEB1 is expressed at control levels, while Vimentin is significantly higher than the control (Fig. 7, OXi4503 treated) as we found in our previous study [9]. Interestingly in the Sunitinib and the combination treatments at day five post treatment while overall E-cadherin staining is lower than the control there is a re-distribution from the cell junctions to the cytoplasm and nucleus of live tumor cells (Fig. 7, Sunitinib and combination treated). ZEB1 expression in the Sunitinib and combination treatments is significantly higher than in the control tumors and displays mainly cytoplasmic localization. The levels of Vimentin are also high in the Sunitinib and combination treatments at day 5 (Fig. 7, Sunitinib and combination treated). Additional file 3 depicts a semi-quantitive enumeration of these results. The results show that ZEB1 expression remains high while the treating agent is present as in the continuous treatments of Sunitinib and the combination while in the single OXi4503 treatment where the agent has been eliminated by day 5, its levels returned to background. Taken together, these results suggest that the tumor cells which survive treatment undergo EMT which lasts while the treating agent is present.

## Discussion

VDA agents like OXi4503 destroy the established tumor vasculature resulting in very significant tumor death [21], however the treatment also induces a robust influx of bone marrow originating cells, including precursor endothelial cells, that home into the surviving tumor areas aiding revascularization [22]. It has been suggested that combinations of VDA with anti-angiogenic drugs could achieve better treatment outcomes. Indeed early studies in animal models demonstrated that an anti-VEGF monoclonal antibody in combination with the OXi4503 treatment prevented mobilization of precursor endothelial cells and resulted in significant tumor reduction [22]. Our study using the anti-angiogenic drug Sunitinib in combination with OXi4503 demonstrated significant reduction in viable tumor and prolonged animal survival compared to either single drug treatment. We used drug concentrations that we or others determined to be tolerated and effective in the mouse species. In the first study Sunitinib was given 2 days before the OXi4503 treatment in order to normalise the tumor vasculature for more efficient delivery of the OXi4503 [3], however the results show the combination treatment to be toxic to the animals. Sunitinib has already been approved for the clinical treatment of certain types of tumors and has been shown to significantly extent patients’ life expectancy in these diseases [23]. Clinical and preclinical reports however have shown that Sunitinib also affects normal vessels and when used as a monotherapy or in combination treatments for long term or in high dosage it is often associated with adverse effects including hypertension, arterial thromboembolic events, renal damage, impaired wound healing, and liver toxicity [24, 25]. El Mesbahi et al. reported hepatic cytolysis in two patients following Sunitinib treatment but reduced Sunitinib dosing returned the patients liver chemistries to normal levels [26]. Our studies also demonstrate that liver toxicity can be ameliorated with shorter exposure to Sunitinib and still maintain combined gain in efficacy. Taken together these results suggest optimisation of dose and/or length of treatment might overcome the toxicity problem in the clinic.

We examined the likely mechanisms that contributed to the increased efficacy of the combination treatment. Revascularization was completely inhibited compared to the single OXi4503 treatment. In agreement with Czabanka et al. our study also demonstrated the ability of Sunitinib to interfere with pre-existing tumor vessels in addition to inhibiting new vessel formation [27]. Interestingly Sunitinib also preferentially destroys the central tumor vessels thus supporting our previous finding that tumor vessels in the periphery are more robust and resistant to treatments [10]. While Sunitinib treatment reduced vessel density compared to control tumors it did not affect some of the larger established tumor vessels and thus it was not as effective in reducing tumor mass as the OXi4503 treatment. The additional efficacy of the combination treatment is due to inhibition of neo-angiogenesis since a significant reduction in tumor vessel density was found compared to either treatment alone. These results are in complete agreement with a recent study by Lee et al. [28] who also demonstrated a significant vessel reduction upon OXi4503/Sunitinib treatment in a subcutaneous human xenograft. Our results in addition demonstrate these effects in an orthotopic CRLM model in an immunologically competent host. We also found significant increase in tumor apoptosis and significant decrease in tumor proliferation, in the combination treatment compared to single arm treatments, thus accounting for the observed reduction in viable tumor. Yang et al. reported that Sunitinib induces medulloblastoma tumor cell apoptosis by inhibiting the STAT3 and AKT pathways [29]. These changes most likely result from increased hypoxia, lack of nutrient availability and ROS accumulation.

Taken together these results indicate promise for testing this combination in the clinic, however there are concerns that suggest additional preclinical work is needed. The first issue that arose from our study is the observed liver toxicity associated with Sunitinib treatment which was exacerbated in the combination treatment. The second issue arising from this study and other published studies is that while the combination treatment increases efficacy and survival, tumor progression ultimately occurs. We used a single maximum tolerated dose of OXi4503 in this study. In other studies it was shown that repeated OXi4503 dosing once tumor vessels re-establish is effective in controlling tumour growth [21]. More recent studies however show that new tumor vessels eventually become resistant to repeat treatments [28]. Similarly AAs whether in single or combination treatments at best prolong survival but tumor resistance and often increased metastasis occur [30]. These findings suggest that while VDA/AA combination treatment could prolong survival, this treatment may be more effective if administered with additional treatment modalities aiming to counteract the development of resistance. In our previous studies we demonstrated a widespread but transient EMT in the tumor cells within the viable rim after OXi4503 treatment. EMT has been shown to be associated with metastasis, and stem cell attributes including tumour resistance to drugs and other therapies [31]. In this study we also demonstrated EMT cell morphology in the Sunitinib and the combination treatments. Interestingly E-cadherin expression while lower than the control did not completely disappear as observed in our previous OXi4503 study but it re-distributed from the cell junctions to the cytoplasm and nucleus, while ZEB1 and Vimentin expression were both high at the termination of the treatment. Nuclear accumulation of E-cadherin has been reported in several types of cancers; oesophageal squamous cell carcinomas, pituitary adenomas and less frequently in colon cancers (6 %) [32, 33] and has been associated with tumor invasion. These results suggest that EMT is a dynamic process responding to changes in the microenvironment. Thus a very acute treatment such as a single OXi4503 treatment, results in immediate loss of E-cadherin and a significant but transient up-regulation of ZEB1 while Vimentin has a longer turnover time [9]. Continuous Sunitinib treatment on the other hand is a less acute treatment and E-cadherin becomes redistributed rather than totally lost, while ZEB1 and Vimentin expression remain high for the duration of the treatment. Nevertheless the surviving tumor cells in both continuous Sunitinib and the combination treatments display EMT morphology that could contribute to therapy resistance and the increased incidence of metastasis associated with Sunitinib treatment [30]. Targeting the mesenchymal tumor cells or preventing the EMT transition in combination to VDA/AA treatment could produce a longer lasting treatment or a total therapy.

## Conclusion

In conclusion, our results demonstrate the potential of VDAs and AAs in combination therapy to treat CRCLM. However, the study also highlights the challenges which need to be addressed before these therapies can be safely and efficiently introduced into the clinic. Careful dose/time regimen needs to be explored for maximal benefit with least adverse effects. Further a treatment regimen consisting of just VDAs and AAs may result in initial regression of the tumor, however surviving tumor cells appear to undergo EMT, making them resistant to treatment and more likely to metastasize to a distant site. These results suggest that VDAs and AAs may exert maximal effects when combined with EMT inhibitors.

## Abbreviations

AA, anti-angiogenic agent; CRLM, colorectal liver metastasis; DMH, dimethyl hydrazine; EMT, epithelial to mesenchymal transition; EPC, endothelial progenitor cells; MoCR, mouse colorectal tumor; VDA, vascular targeting agent

## Additional files

Additional file 1: Effects of treatment on tumor. Tumor death is evaluated with H&E staining. Live tumor areas are enclosed within double lines. A single OXi4503 treatment has an immediate effect on tumor death reaching a maximum at 24 h. By day five there is regrowth of the tumor into the necrotic centre. Continous Sunitinib treatment has a gradual reducing effect on live tumor but it does not reach the levels of tumour killing seen by the OXi4503 treatment at 24 h. (PDF 381 kb) Additional file 2: OXi4503, Sunitinib and combination treatments induce EMT in the surviving tumor cells. Formalin-fixed control and treated tumor sections were stained with antibodies to E-cadherin, ZEB1, or Vimentin. Positive expression is detected by the brown staining. Scale bar = 200 mm. L = liver, T = live tumor. NT = necrotic tumor. Images are representative for each treatment group (n ≥ 5 animals). (PDF 417 kb) Additional file 3: Intensity score of EMT changes in tumor metastases following 5 days of OXi4503, Sunitinib and combination treatments. E-cadherin expression decreased in all treatment groups but only reached significance in the OXi4503 treatment (**P* = 0.005). ZEB1 expression significantly increased in the Sunitinib and Sunitinib/OXi4503 combination treatments (**P* < 0.025). Vimentin expression revealed a significant increase following OXi4503 treatment (**P* = 0.037). (PDF 249 kb)
