# Supplementary material for: Vascular disruptive agent OXi4503 and anti-angiogenic agent Sunitinib combination treatment prolong survival of mice with CRC liver metastasis
Source: BMC Cancer. 2016 Jul 26;16:533. doi: 10.1186/s12885-016-2568-7 (PMC4962549; doi:10.1186/s12885-016-2568-7)
Supplement: Additional file 1: — Effects of treatment on tumor. Tumor death is evaluated with H&E staining. Live tumor areas are enclosed within double lines. A single OXi4503 treatment has an immediate effect on tumor death reaching a maximum at 24 h. By day five there is regrowth of the tumor into the necrotic centre. Continous Sunitinib treatment has a gradual reducing effect on live tumor but it does not reach the levels of tumour killing seen by the OXi4503 treatment at 24 h. (PDF 381 kb) [file 12885_2016_2568_MOESM1_ESM.pdf]

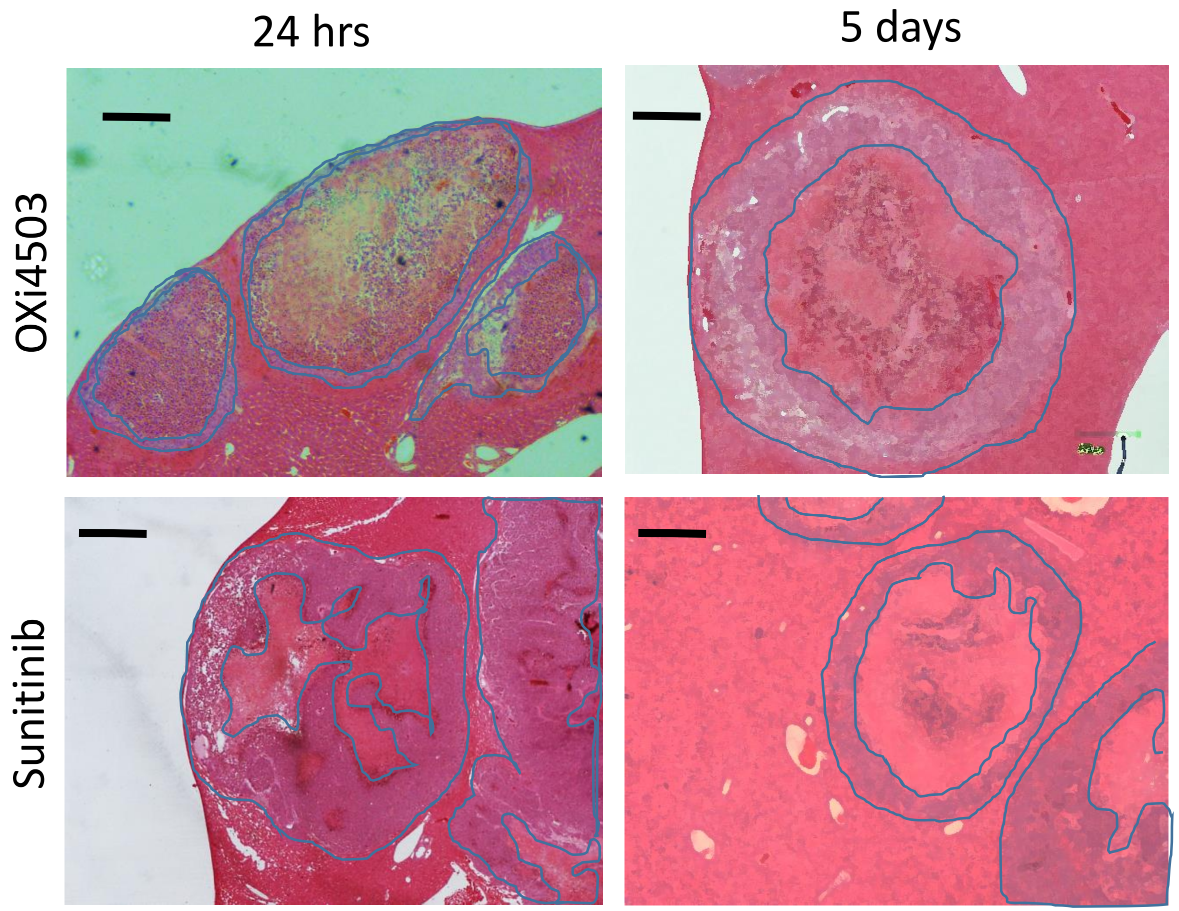

Additional File 1: Effects of treatment on tumor. Tumor death is evaluated with H&E staining. Live tumor areas are enclosed within double lines. A single OXi4503 treatment has an immediate effect on tumor death reaching a maximum at 24 hrs. By day five there is regrowth of the tumor into the necrotic centre. Continuous Sunitinib treatment has a gradual reducing effect on live tumor but it does not reach the levels of tumour killing seen by the OXi4503 treatment at 24 hrs.
