# Supplementary material for: Vascular disruptive agent OXi4503 and anti-angiogenic agent Sunitinib combination treatment prolong survival of mice with CRC liver metastasis
Source: BMC Cancer. 2016 Jul 26;16:533. doi: 10.1186/s12885-016-2568-7 (PMC4962549; doi:10.1186/s12885-016-2568-7)
Supplement: Additional file 2: — OXi4503, Sunitinib and combination treatments induce EMT in the surviving tumor cells. Formalin-fixed control and treated tumor sections were stained with antibodies to E-cadherin, ZEB1, or Vimentin. Positive expression is detected by the brown staining. Scale bar = 200 mm. L = liver, T = live tumor. NT = necrotic tumor. Images are representative for each treatment group (n ≥ 5 animals). (PDF 417 kb) [file 12885_2016_2568_MOESM2_ESM.pdf]

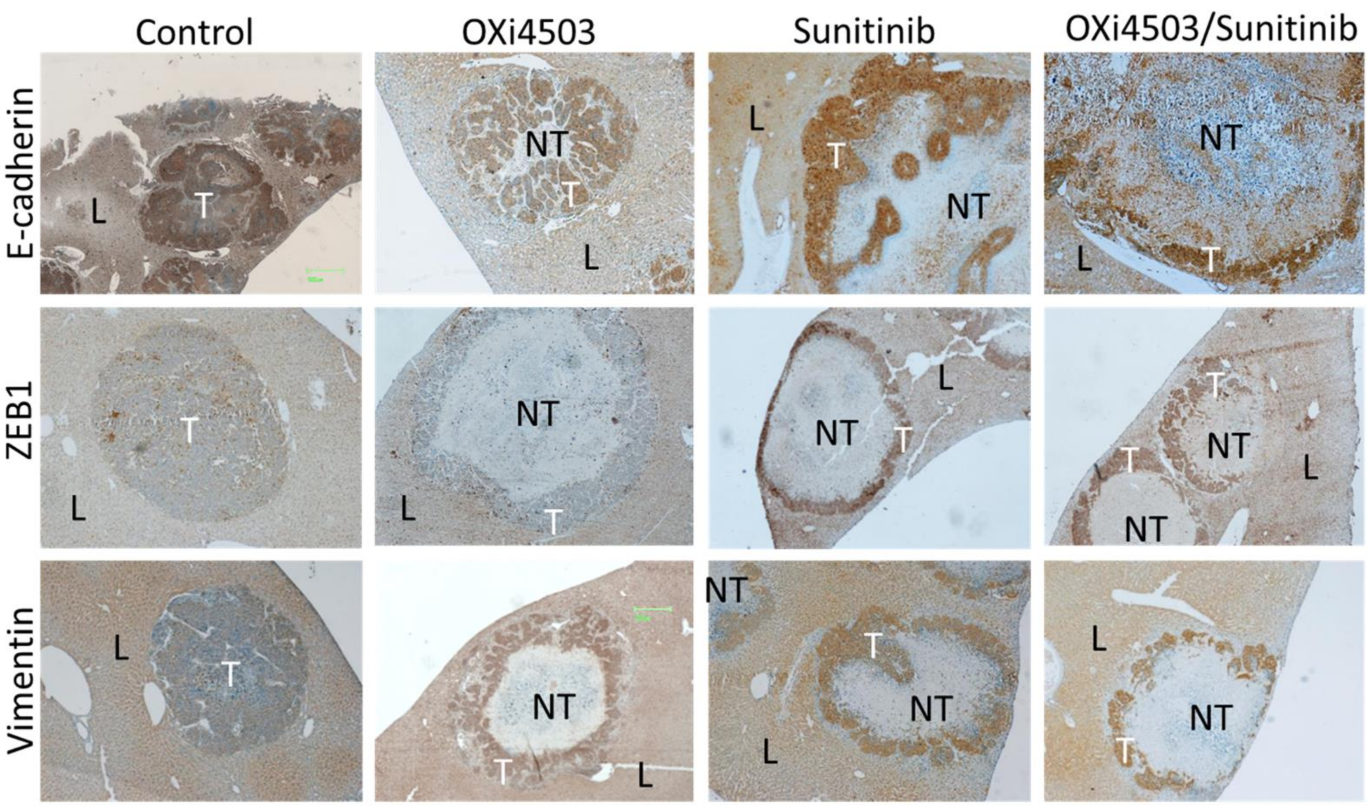

Additional File 2: OXi4503, Sunitinib and combination treatments induce EMT in the surviving tumor cells. Formalin-fixed control and treated tumor sections were stained with antibodies to E-cadherin, ZEB1, or Vimentin. Positive expression is detected by the brown staining. Scale bar=200 mm. L=liver, T= live tumor. NT= necrotic tumor. Images are representative for each treatment group (n≥5 animals).
