# Supplementary material for: Vascular disruptive agent OXi4503 and anti-angiogenic agent Sunitinib combination treatment prolong survival of mice with CRC liver metastasis
Source: BMC Cancer. 2016 Jul 26;16:533. doi: 10.1186/s12885-016-2568-7 (PMC4962549; doi:10.1186/s12885-016-2568-7)
Supplement: Additional file 3: — Intensity score of EMT changes in tumor metastases following 5 days of OXi4503, Sunitinib and combination treatments. E-cadherin expression decreased in all treatment groups but only reached significance in the OXi4503 treatment (*P = 0.005). ZEB1 expression significantly increased in the Sunitinib and Sunitinib/OXi4503 combination treatments (*P < 0.025). Vimentin expression revealed a significant increase following OXi4503 treatment (*P = 0.037). (PDF 249 kb) [file 12885_2016_2568_MOESM3_ESM.pdf]

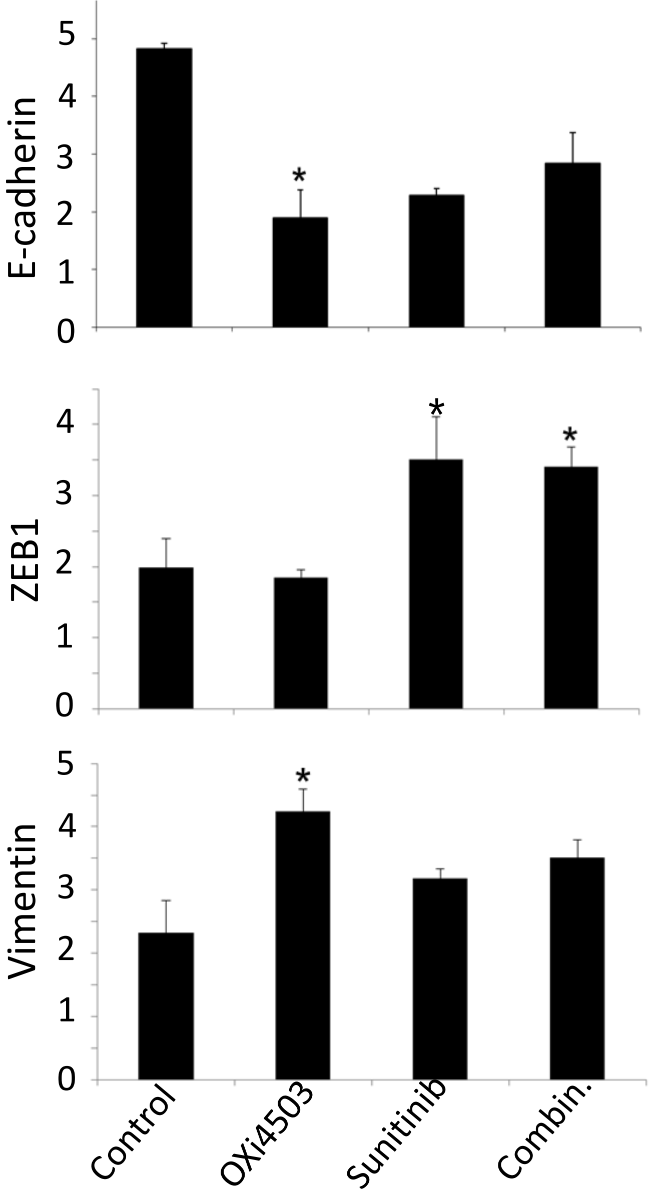

Additional File 3: Intensity score of EMT changes in tumor metastases following five days of OXi4503, Sunitinib and combination treatments. E-cadherin expression decreased in all treatment groups but only reached significance in the OXi4503 treatment (*P=0.005). ZEB1 expression significantly increased in the Sunitinib and Sunitinib/OXi4503 combination treatments (*P<0.025). Vimentin expression revealed a significant increase following OXi4503 treatment (*P=0.037).
